# Dynamic Functional Connectivity as a complex random walk: Definitions and the dFCwalk toolbox

**DOI:** 10.1016/j.mex.2020.101168

**Published:** 2020-12-01

**Authors:** Lucas M. Arbabyazd, Diego Lombardo, Olivier Blin, Mira Didic, Demian Battaglia, Viktor Jirsa

**Affiliations:** a Université Aix-Marseille, INSERM UMR 1106, Institut de Neurosciences des Systèmes, F-13005 Marseille, France; b AP-HM, Timone, Service de Pharmacologie Clinique et Pharmacovigilance, F-13005 Marseille, France; c AP-HM, Timone, Service de Neurologie et Neuropsychologie, F-13005 Marseille, France

**Keywords:** Neuroimaging, fMRI, Functional Connectivity, Chronnectome

## Abstract

• We have developed a framework to describe the dynamics of Functional Connectivity (dFC) estimated from brain activity time-series as a complex random walk in the space of possible functional networks. This conceptual and methodological framework considers dFC as a smooth reconfiguration process, combining “liquid” and “coordinated” aspects. Unlike other previous approaches, our method does not require the explicit extraction of discrete connectivity states.
• In our previous work, we introduced several metrics for the quantitative characterization of the dFC random walk. First, dFC speed analyses extract the distribution of the time-resolved rate of reconfiguration of FC along time. These distributions have a clear peak (typical dFC speed, that can already serve as a biomarker) and fat tails (denoting deviations from Gaussianity that can be detected by suitable scaling analyses of FC network streams). Second, meta-connectivity (MC) analyses identify groups of functional links whose fluctuations co-vary in time and that define veritable dFC modules organized along specific dFC meta-hub controllers (differing from conventional FC modules and hubs). The decomposition of whole-brain dFC by MC allows performing dFC speed analyses separately for each of the detected dFC modules.
• We present here blocks and pipelines for dFC random walk analyses that are made easily available through a dedicated MATLAB^Ⓡ^ toolbox (*dFCwalk*), openly downloadable. Although we applied such analyses mostly to fMRI resting state data, in principle our methods can be extended to any type of neural activity (from Local Field Potentials to EEG, MEG, fNIRS, etc.) or even non-neural time-series.

## Graphical abstract

**Figure:**
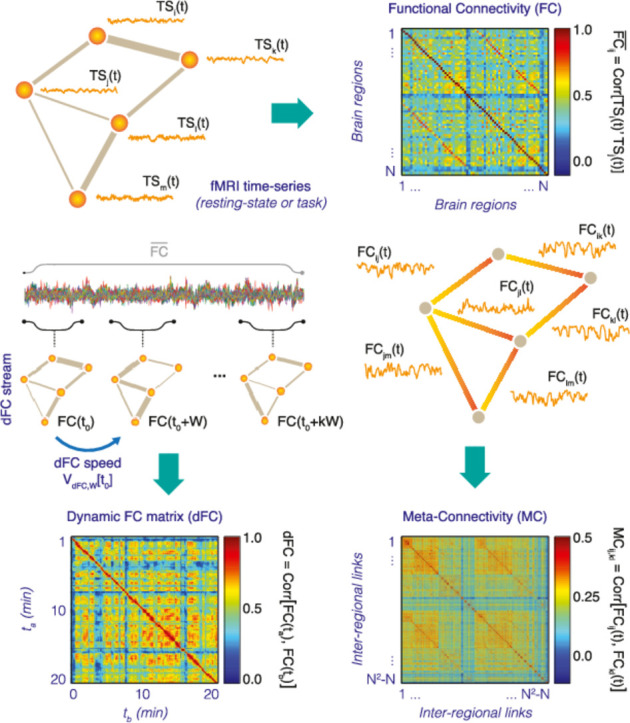

Specifications table

**Table:** 

Subject Area:	Neuroscience
More specific subject area:	*Neuroimaging; functional connectomics*
Method name:	*dFC random walk analyses*
Name and reference of original method:	• *Battaglia, D., Boudou, T., Hansen, E.C.A., Lombardo, D., Chettouf, S., Daffertshofer, A., Mcintosh, A.R., Zimmermann, J., Ritter, P., Jirsa, V., 2020. Dynamic Functional Connectivity between order and randomness and its evolution across the human adult lifespan. NeuroImage 222, 117,156. doi:10.1016/j.neuroimage.2020.117156 and* • *Lombardo, D., Cassé-Perrot, C., Ranjeva, J.-P., Le Troter, A., Guye, M., Wirsich, J., Payoux, P., Bartrés-Faz, D., Bordet, R., Richardson, J.C., Félician, O., Jirsa, V., Blin, O., Didic, M., Battaglia, D., 2020. Modular slowing of resting-state dynamic Functional Connectivity as a marker of cognitive dysfunction induced by sleep deprivation. NeuroImage 222, 117,155. doi:10.1016/j.neuroimage.2020.117155* • *Hansen, E.C.A., Battaglia, D., Spiegler, A., Deco, G., Jirsa, V., 2015. Functional connectivity dynamics: modeling the switching behavior of the resting state. NeuroImage 105, 525–535. doi:10.1016/j.neuroimage.2014.11.001.*
Resource availability:	*The dFCWalk toolbox can be downloaded at the link:*https://github.com/FunDyn/dFCwalk.git*under the Creative Commons Zero v1.0 Universal license*

## Method details

While *structural connectivity* refers to the existence of an anatomical connection between two neuronal regions –with the compilation of structural links between all pairs of anatomically interconnected regions known as the *connectome* [44]–, the notion of *functional connectivity* (FC) intends to capture the existence of coordinated activity between two brain network nodes. Therefore, functional connectivity is necessarily way more flexible than the underlying structural connectome [37], being potentially the manifestation of collective emergent dynamics [9,30]. Notably, during the resting state (rs; [21]), the flexible sampling of a rich repertoire of possible dynamical states compatible with a given connectome [18,23] is expected to lead to a complex reconfiguration of FC along time, the characterization of which is the endeavor of the nascent field of “chronnectomics” [12]. Indeed, the brain is restless even at rest and rs FC continually fluctuates in a way which displays a nonrandom spatiotemporal organization [22]. There is growing evidence that such *dynamic Functional Connectivity* (dFC), in both rest and task conditions, can be used as a sensitive biomarker of the efficiency and flexibility of cognitive processing [7,^14^,43] as well as of pathological alterations of resting state dynamics [8,^15^,28].

To the growth in the number of dFC studies, has corresponded an analogous inflation in the number of possible methods to extract and quantitatively parameterize dFC from fMRI data or other time-series of neural activity (such as EEG, MEG, fNIRS…). We invite the reader to refer to e.g. Preti et al. [40] for a non-exhaustive review. Many approaches have tried to characterize “states of FC” that would be visited at different times by the evolution of FC. State extraction has been achieved, e.g., by direct clustering of instantaneous FC networks observed in different temporal windows [2], by temporal network approaches [45] or by fitting of statistical generative models of connectivity states and state transitions [3,48]. However, statistical evidence in favour for the actual non-stationarity of rs FC [25,51] or the actual existence of well-distinct FC states [42] is not completely conclusive. A problem with state-extraction-based dFC methods is indeed that, by assuming implicitly the existence of FC states, they will find them even when a description of dFC in terms of discrete states is not fully pertinent.

In Battaglia et al. [5] and Lombardo et al. [34] we have introduced a novel methodological framework that circumvents the problem of detecting FC states or assessing stationarity or non-stationarity of dFC. In this new framework –which we here refer to as *dFCwalk* paradigm–, we conceptualize dFC as a smooth reconfiguration flow across continually morphing FC configurations. In the dFCwalk vision, an instantaneously observed FC network is seen as a “point” in the space of possible FC network realisations. By evolving in time, this point performs a stochastic exploration of the high-dimensional space of possible FC, describing a veritable random walk. In the dFCwalk paradigm, dFC properties are quantified as descriptors of the dFC random walks implemented by collective brain dynamics during a specific recording or imaging session. Each session is indeed mapped to a different path in FC space. Relatively small variations of FC from one observation time to the next will result in short flight lengths and more extensive network reconfigurations in larger flight lengths. By keeping the interval between two consecutive FC network time-resolved estimations constant, shorter and longer flight lengths can also naturally be reinterpreted as associated to a slower or faster *speed of dFC reconfiguration*. A first aim of the dFCwalk analysis framework will be therefore to suitably quantify and describe distributions of dFC speed (notably, their mode, giving a *typical dFC speed*, and their spread).

Beyond the speed at which dFC travels along a walk, it will be also important, as a second aim, to describe the general geometry of the dFC paths. Random walks giving rise to stochastic paths, a natural way to characterize their “shape” will be to measure their *fractal scaling properties.* Indeed the fractal geometries of stochastic paths will be different, depending on the degree of correlation between the flight lengths travelled at consecutive times [36], varying from “memoryless” (as in Brownian motion) to positive or negative sequential autocorrelations (resulting in non-Gaussian stochastic processes, such as Levy walks). A tool suitable for the scaling analysis of random walk paths is Detrended Fluctuation Analysis (DFA; [39,^46^,50]), which we have adapted to dFC walks in Battaglia et al. [5]. Such analysis provides an estimation of the fractal geometry of dFC paths, in terms of a DFA exponent, α_DFA_ = 0.5 for memoryless walks, and α_DFA_ > 0.5 (α_DFA_ < 0.5) for walks with positive (negative) sequential correlations in flight lengths.

Exactly as typical static (time-averaged) FC analysis ignore time, the previously mentioned dFCwalk analyses introduced in Battaglia et al. [5] ignore space. However, FC reconfiguration may occur at different speeds for different sets of links. Furthermore, the fluctuations of certain FC links may covary with the fluctuation of other FC links –forming a *dFC module*– but be relatively independent from the fluctuation of other sets of links. Therefore, it may make sense to compute a different dFC speed distribution for different dFC modules (*modular dFC speeds*), since each of these modules performs its own specific random walk. A third aim of the dFCwalk framework will thus be the identification of these eventual dFC modules fluctuating in parallel according to independent random walks. To do so, we have introduced in Lombardo et al. [34] and edge-covariance-based method which we called *Meta-Connectivity* (MC) analysis, and which is strongly related (although not identical) to other edge-centric functional connectivity approaches introduced elsewhere [4,^10^,20]. In this approach, dFC modules are extracted applying conventional modular decomposition algorithms –as the Louvain method– to the MC matrix. Indeed, if we consider FC links as nodes of a new network, dual (see e.g. [6] for the notion) to the original FC network, the MC matrix entries can be interpreted as “links between links”, or, as we say, *meta-connections*. In this vision, MC is still a graph, although over a broader set of nodes than the original FC graph and any usual graph-theoretical method valid for standard networks can also be applied to MC. As discussed by Lombardo et al. [34], links which are strongly covarying tend to be incident to a common region. Therefore, dFC modules tend to include star subgraphs with well-defined center regions –*meta-hubs*– and divergent links which tend to be all strong or all weak simultaneously depending on time. These meta-hubs act as “puppet masters” moving the “threads” in specific dFC modules and reaching to regions which can be far away and potentially distributed brain-wide. Therefore, meta-hubs are localized controllers of non-localized functional systems and are in this sense very different from conventional FC hubs (see [34] for a detailed discussion).

Altogether, in the two manuscripts by Battaglia et al. [5] and Lombardo et al. [34] we have forged a rich toolbox of interdependent analyses and metrics that have proved to be able to track changes of dFC properties across aging or in relation with variations of cognitive performance. We have now made this toolbox available in the form of a collection of MATLAB^Ⓡ^ functions (dFCwalk toolbox, download link: https://github.com/FunDyn/dFCwalk.git, under Creative Commons Zero v1.0 Universal license (CC0)) that allow coding complex analysis designs such as the ones of Battaglia et al. [5] and Lombardo et al. [34] with just a few lines of high-level code. We describe here in the following in a step-by-step way how to implement the various dFC random walk analyses we have mentioned, providing specific example applications for illustration.

### The input needed for dFCwalk analyses

The set of methods which we will review here can in principle be applied to whatever type of multivariate time-series. The examples here included are resting state BOLD time-series from fMRI experiments, however precisely the same analyses (with parameters, such as window sizes, adjusted to the specificities of the signals of interest) may be applied to EEG or MEG or whatever other neuronal activity time-series. As a matter of fact, in Clawson et al. [13] and Pedreschi et al. [38] we have presented very related analyses performed on spiking activity raster plots or multichannel local field potentials. Even more generally, such analyses may be used for non-neural multivariate time-series, as, e.g. EMGs [47] or functional connectivity analyses in geosciences [49].

We define therefore as input a set of time-series *TS_i_(t)* with *i* = 1…*N* and 0 ≤ *t* ≤ *T* where *N* is the number of network nodes (brain regions, voxels or channels for neuroscience datasets) and *T* is the number of time samples in the time-series.

### Functional connectivity and dFC stream

Once given a set of time-series *TS(t)*, it is possible to compute the time-averaged (static) Functional Connectivity *(FC)* matrix, with entries:

$${\bar{FC}}_{ij}=\text{Corr}\left[TS_{i}\left(t\right),TS_{j}\left(t\right)\right],\text{with}\;0\leq t\leq T$$

**Fig. 1:**
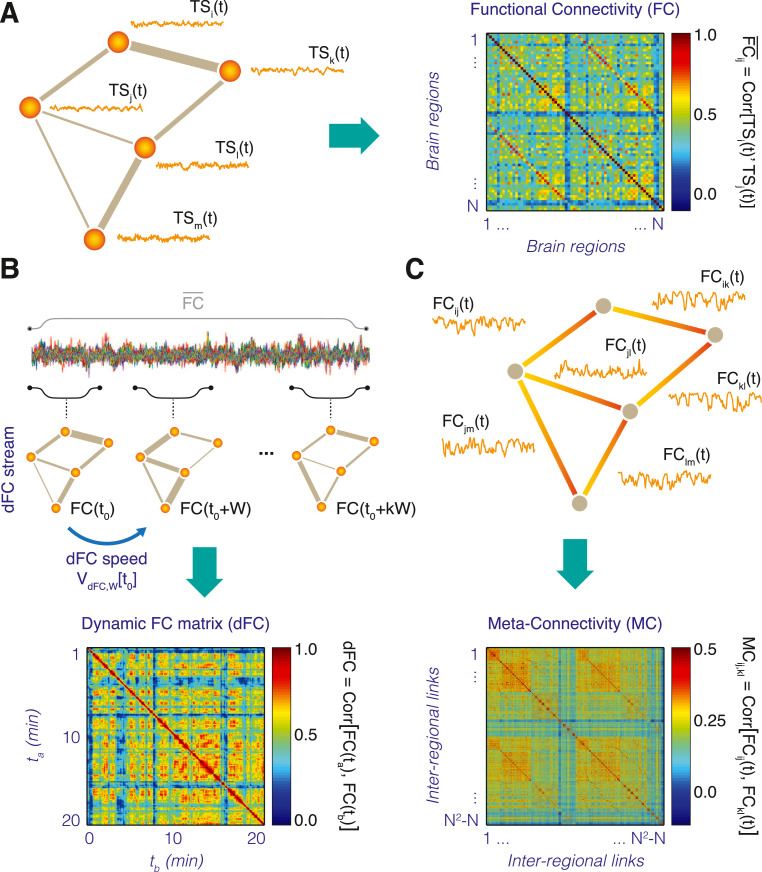
*From Functional Connectivity to Functional Connectivity Dynamics.* (A) Traditionally, correlations between neural activity time-series *TS_i_(t)* of *N* different brain region nodes *i* and *j* (left) are averaged over long times and compiled into the entries FC*_ij_* of a ‘static’ *N-*times-*N* Functional Connectivity (FC) matrix (right). (B) Sliding windows of a shorter temporal duration, it is possible to estimate a stream of time-resolved FC*(t)* networks, which we call the *dFC stream* (top). The variation between a FC frame at a time *t_0_* and the next non-overlapping frame at a time *t_0_* + *W* is measured by the *dFC speed V_dFC,W_(t_0_)* where *W* is the chosen window size. The degree of similarity (inter-matrix correlation) between FC*(t)* networks observed at different times is then represented into a *F*-times*-F* recurrence matrix, or *dynamic Functional Connectivity* (dFC) matrix, where F*T* is the total number of probed windows (i.e. frames in the temporal network given by the dFC stream), depending on window size and overlap (bottom). (C) Alternatively, one can consider each individual FC link as a dynamic variable FC*_ij_(t)* attached to the graph edge between two regions *i* and *j* (top). Generalizing the construction of the FC matrix in panel (A), we can thus extract a *N(N-1)-*times-*N(N-1)* matrix of covariance between the time-courses of different FC*_ij_(t)* links. We re-baptized this inter-link covariance matrix as *Meta-Connectivity* (MC) matrix.

This matrix gives the normalized pairwise Pearson correlation between time-series, averaged over the total session time *T* (Fig. 1A). Note that all entries are retained in the matrix, independently of whether the correlation values are significant or not or without fixing any threshold. This is because we consider FC entries (and other features in general in the following) as “features” that can be useful for tracking trends or separating cohorts, i.e. we compute them for their potential predictive value more than for their assessment itself.

In the dFCwalk toolbox the function to compute a static FC matrix out of time-series *TS* is TS2FC, taking as input a generic *TS* matrix variable (different columns are different regions, different rows are different times) and producing as output the static FC entries (in either ‘matrix’ or ‘vector’ format, see later).

To generate not a single time-averaged matrix of FC but an entire stream of FC matrices evaluated at different times along the session –i.e. a temporal network [27,32], called the “*dFC stream”*–, the dFCwalk toolbox uses a sliding window approach (Fig. 1B, top). Individual temporal frames of the dFC stream are evaluated using the same formula as above, restricted to the considered time-window:

$$FC_{ij}\left(t_{k}\right)=\text{Corr}\left[TS_{i}\left(t\right),TS_{j}\left(t\right)\right],\text{with}\;t_{k}\leq t\leq t_{k}+W$$

where *t_k_* is the start time of the *k*-th temporal frame of the network and *W* is a fixed observation window length. Frame start times are separated by a sliding step ∆τ, so that *t_k_* = t_k-1_ *+* ∆τ = *k*∆τ. This sliding increment is often fixed to be equal to the window size *W* so that consecutive network frames are evaluated on non-overlapping time-series segments, however it can be fixed to a value of choice. Typically, we used ∆τ = *W* for dFC speed distribution analyses, but set it to values as small as ∆τ = 1 time-step in the time-series (e.g. one TR for fMRI time-series) for analyses requiring more continuous dFC streams such as DFA or MC analyses. In the dFCwalk toolbox the function to compute an entire dFC stream out of time-series *TS* is TS2dFCstream, taking as input a generic *TS* matrix variable, a window size *W* and, optionally, a sliding increment ∆τ (which is otherwise fixed as default at ∆τ = *W*). The output is the temporal network itself.

Both TS2FC and TS2dFCstream can produce outputs in two different formats. The FC matrices are symmetric and therefore, despite their size is *N* × *N*, they just have *L = N(N-1)*/2 independent entries, where L is the number of links, corresponding to the lower triangular part of the matrix. When using the ‘matrix’ format (or ‘2D’, default for TS2FC) the output FC matrix (or each of the dFC stream frames) are formatted as actual *N* × *N* matrices. Therefore, the output of TS2dFCstream, in matrix format, will be a 3D tensor of size *N* × *N* × *F*, where *F* is the number of frames produced given the actual choices of *W* and ∆τ (frames with windows with trimmed length that may occur at the terminal part of the time-series are dropped). In ‘vector’ format (or ‘1D’ for TS2FC, and therefore ‘2D’ default for TS2dFCstream), on the contrary, only the independent entries of FC or of each FC frame will be provided as output, so that the output of TS2FC will be a vector of size *L* × 1 and the output of TS2dFCstream a 2D matrix of size *L* × *F* (each of the *F* frames being given in vector format). The vector format has the advantage of producing smaller output variables occupying less space in memory, since dFC stream variables can be quite large for very long time-series and large numbers of nodes. It has also the advantage that FCs in ‘vector’ format are naturally formatted as ‘points’ in a vector space which makes more straightforward the generation of dimensionally reduced representations of the dFC stream random walk via projection methods, such as e.g. t-stochastic neighbourhood embedding [26], accessible in the standard MATLAB^Ⓡ^ Statistical toolbox via the function tsne.

For the sake of computing speed, all computations are always performed in the compact ‘vector’ format even when inputs are provided in ‘matrix’ format (an automatic conversion to ‘vector’ format is performed by the dFCwalk toolbox functions). The dFCwalk toolbox functions Matrix2Vec can be called to perform conversions from 2D FC matrices in *N* × *N* matrix format to 1D vector FCs of size *L* × 1 or from 3D dFC streams in *N* × *N* × *F* tensor format to 2D dFC streams of size *L* × *F*.

### Recurrence analysis via the dFC matrix

After extracting a dFC stream, it is possible to study its recurrence structure, verifying whether the FC*(t)* networks observed at certain times are transiently stabilized and possibly return in different epochs or whether there are special transients of anomalously fast network reconfiguration. To do so, one can study the similarity between FC frames observed at different times, evaluating the so-called *dynamic Functional Connectivity (dFC) matrix* of a dFC stream. We introduced this matrix for the first time in Hansen et al. [23], but it constitutes in reality just an example of recurrence analysis, common in time-series analyses [29], here adapted to temporal networks. To measure the similarity between two networks FC*(t_1_)* and FC*(t_2_)* we use plain correlation between the upper triangular parts of the two matrices. The entries of the dFC matrix are then given by:

$$dFC\left(t_{1},t_{2}\right)=\text{Corr}\left[UpperTri\left(FC\left(t_{1}\right)\right),UpperTri\left(FC\left(t_{2}\right)\right)\right]$$

**Fig. 2:**
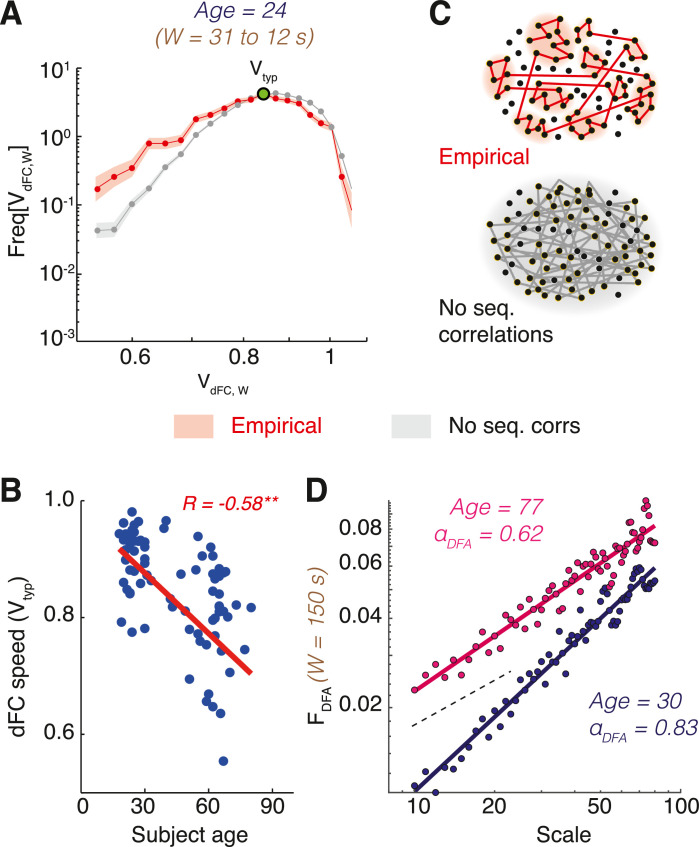
*dFC speed distributions and long-range correlations.* (A) Distributions of resting state fMRI dFC speed, shown here for a representative subjects (log-log scale, pooled window sizes 12 *s* ≤ *W <* 31 s) displayed a peak at a value *V_typ_* (*typical dFC speed*) and a fat left tail, reflecting an increased probability with respect to chance level to observe short dFC flight lengths (95% confidence intervals are shaded: red, empirical; gray, chance level for shuffled surrogates, see Fig. 4). (B) The dFC speed *V_typ_* decreased with subject age (results are shown here for the specific window pooling used in panel A, but robust for other choices as well; cf. [5] for details). The FC space was seemingly explored through an anomalous random process in which short steps were followed by short steps with large probability (sequential correlations), leading to “knotted” trajectories (panel C, top). This contrasts with a standard random process, visiting precisely the same FC configurations but without long-range correlations (panel C, bottom). (D) The presence of long-range sequential correlations (persistence) of dFC could be proved through a Detrended Fluctuation Analysis (DFA) adapted for dFC streams. We show here DFA log-log scaling plots for representative subjects (in the “young” group, in blue; or in the “older” group, in magenta) and a representative window sizes (*W =*  150 s, in the middle of a broad range in which persistence is displayed, see [5]). The linearity of DFA scatter plots on the log-log plane (scale of coarse-graining vs total fluctuation strength) reveals that instantaneous increments along the dFC stream form a self-similar sequence. The black dashed lines indicate the slope that would be associated to α_DFA_ = 0.5, i.e. the case of ordinary uncorrelated Gaussian random walk. Linear slopes are steeper than for ordinary random walk, indicating that dFC streams evaluated at this window size (and, generally, at *W* > ~20 s) follow a persistent stochastic walk.

One could however redefine the analysis to use any other more sophisticated metric of network similarity (e.g. Jaccard similarity coefficient in Pedreschi et al. [38]). Note that the obtained dFC matrix depends on the window-size W and the sliding step ∆τ adopted to evaluate the dFC stream. As shown in the example of Fig. 1B (bottom), dFC matrices for resting state fMRI sessions usually display characteristic patterns composed out of square-shaped red-hued blocks, corresponding to epochs of transiently increased similarity between consecutive FC(t) network frames. Such epochs of relative FC stability increase –or ‘*dFC knots*’– are intertwined with transients of relative instability, appearing as light blue stripes in the dFC matrix, denoting strong dissimilarity from previously visited FC*(t)* networks. During such transients –or ‘*dFC leaps*’– FC(t) quickly morphs before stabilizing again into the next dFC knot. As discussed in Battaglia et al. [5], this alternation between knots and leaps (cf. also Fig. 2) can be seen as the existence of a “liquid clustering” in dFC streams, where sharply separated FC states do not exist, but epoch of transient stabilization into alternative FC configurations can be nevertheless be detected. Longer and more persistent “knots” are observed for increasing age [5] or after Sleep Deprivation [34]. In Clawson et al. [13] or Pedreschi et al. [38] where we have conducted similar analysis (using Jaccard similarity coefficient as network similarity measure) on neural activity at the micro-scale within hippocampal formation local circuits, dFC analyses led on the contrary to detect sharp blocks, corresponding to the existence of a spectrum of properly discrete connectivity states (with large internal variability, though).

In the dFCwalk toolbox, one can compute a dFC matrix from a dFCstream variable (in either the 3D ‘tensor’ or the 2D ‘matrix’ formats) using the function dFCstream2dFC. This function, which does not require any other input argument, always produces a *F* × *F* matrix, where *F* is the number of network frames in the input dFC stream. To modify the used window size *W* or sliding step (lag) ∆τ one must thus recompute the dFC stream and feed it into dFCstream2dFC, once recomputed with the desired parameters.

### Global dFC speed analysis

The dFC recurrence matrix just provides a visualization of the temporal structure of the dFC stream. For quantitative characterizations one needs to evaluate the rate of FC variation along time. By measuring the distance between two FC observations separated by a fixed amount of time set to be equal to the window-size *W* we thus define the instantaneous *global dFC speed* (cf. Fig. 1B) as:

$$V_{\text{dFC},W}\left(t\right)=1-\text{dFC}\left(t,t+W\right)$$

This dFC speed is termed “global” because evaluated in terms of comparisons of whole-brain FC matrices. This definition makes, once again, dFC speed dependent on the chosen window size *W*. A histogram of dFC speed for a representative subject is shown in Fig. 2A. Various features of interest could be extracted by these histograms such as the position of their mode (*typical global dFC speed)* or measures of dispersion, such as dFC speed variance or interquartile range or median absolute dispersion. All these features could display statistical differences across conditions or subjects. For instance, in Battaglia et al. [5] typical global dFC speed correlated with subject age (cf. here Fig. 2B).

In the case of fMRI sessions, time-series are often short, so that the number of speeds that can be measured is small. To increase the number of dFC speed values and build smoother histograms one can adopt several strategies. The first is *window oversampling*, which is possible when the dFC stream is computed with a sliding step ∆τ smaller than sliding window size *W.* In this case one can get multiple speed estimates from a same time epoch. Indeed one can get a value comparing FC*(t)* measured over the [*t, t* + *W*] interval but also over the [*t +* ∆τ*, t* + ∆τ *+ W*], [*t + 2*∆τ*, t* + 2∆τ *+ W*], … [*t +p*∆τ*, t* + *p*∆τ *+ W*] intervals, as long as (*t* + *p*∆τ) < (*t* + *W*)*.* While these oversampled values do not provide statistically independent samples, still they can help denoising the estimate of instantaneous dFC speed over the [*t, t* + 2 *W*] range.

The second strategy is *window pooling*. The dFC speeds evaluated for a window size *W* and for a second window size *W’* = *W* + ∆*w* of a length close but not commensurate to *W* may have statistically indistinguishable or, at least, very similar and largely overlapping distributions. In this case it is reasonable to merge the list of speed observations evaluated at window size *W* with the list of speeds evaluated at window size *W’* to build a unified histogram for both the window sizes (*W, W’*) pooled together. Once again, window pooling should be taken as a pragmatic operation performed to smooth dFC speed histograms without real rigorous ground. Beyond smoothing histograms, window pooling also allows reduce the number of independent multiple comparisons to perform when sample size is small –as in Lombardo et al. [34]– and to blur eventual spurious peaks of dFC speed resulting from artefactual aliasing due to fixed-size window filtering (cf. [31]). Concretely, there are no rules for choosing which window size to pool together and the scientist must take a decision which is ultimately arbitrary and dependent on dataset and study needs. Therefore, a pooled windows analysis should always be validated by a set of single-window analyses to show that the results of interest hold tendentially also for a majority of individual windows over the range of window sizes pooled together. This is, for instance, what we did in Supporting Figures of both Battaglia et al. [5] and Lombardo et al. [34].

The dFCwalk toolbox provides a high-level function dFC_Speeds, which evaluates single window dFC speeds but is conceived to facilitate window oversampling and pooling. This function produces as first output the typical dFC speed and as second optional output the entire list of instantaneous speeds along the session time-series. To produce these outputs, it takes as input a dFC stream (in either 2D or 3D format) and a second additional argument ∆*f* indicating the “number of steps to the frame to compare” in evaluating speeds. Let us clarify. The default value for this optional parameter is ∆*f* = 1. This means that dFC speeds are computed comparing each frame in the input dFC stream with the next frame in the stream, i.e. the *f*-th frame with the *(f+1)*-th frame. This ∆*f* = 1 choice (default for dFC_Speeds) is particularly suitable to the case of a dFC stream evaluated with ∆τ *= W* (default for dFCstream2dFC). In this case, the straight use of dFC_Speeds and dFCstream2dFC in sequence with their default parameters will produce a single-window dFC speed estimation without window oversampling and window pooling. For instance, the syntax:

[typSpeed,Speeds]=dFC_Speeds(dFCstream2dFC(TS,W))

will directly generate a typical dFC speed typSpeed and a full list of instantaneous dFC speeds Speeds estimates starting from input time-series TS and for a given window size W. To obtain an estimate of typical speed and speed list improved by window oversampling, a small syntax modification is sufficient. The starting point is a dFC stream with a smaller sliding step and, consequently, a larger number of frames (with larger partial overlap). In this case, when evaluating dFC speeds the “number of steps to the frame to compare” will not be any more ∆*f* = 1 because the next frame is overlapping. One will need therefore to feed a larger ∆*f* to ensure that each network frame is compared with a frame evaluated over the next non overlapping time interval. A typical choice may be for instance to adopt the smallest possible sliding step ∆τ *=* 1. Thus, the “number of steps” to the first non-overlapping frame will be ∆*f* = *W*. A window oversampled estimate of speeds will then be obtained in this case using the (slightly more complex) syntax:

[typSpeed,Speeds]=dFC_Speeds(dFCstream2dFC(TS,W,1),W)

Window pooling is also very simple. In this case, one will have to generate and store a temporary Speeds list for each of the windows size separately in the range of windows to pool and then concatenate all these lists into a common list (e.g. PooledSpeeds = [Speeds_W1; Speeds_W2; … Speeds_Wk]). The median of this combined list PooledSpeeds will correspond then to the pooled typical speed for the chosen pooled range (W1,W2,… Wk).

Finally, the dFCwalk toolbox also provides a function for the simple evaluation of dFC speed histograms. The function BuildSpeedHistogram takes as compulsory input a list of instantaneous dFC speed observations (e.g., either a single window Speeds or a pooled windows PooledSpeeds) and plots a histogram of the distribution, with suitable bin-by-bin confidence intervals based on the Agresti and Coull binomial approximation [1]. Additional input and output arguments can be given to control the number and centers of bins used for histogram construction, or to output the numeric values of bin counts and confidence intervals for further statistical analyses.

### Detrended fluctuation analysis of dFC streams

The “shape” of the dFC random walk depends on the sequential ordering of short and long flight lengths, i.e., of slow and fast dFC speeds evaluated at a fixed window size (cf. cartoon in Fig. 2C). To evaluate the presence of autocorrelations in the sequence of speeds and concluding for the presence of memory (short/long steps tend to follow short/long steps), “anti-memory” (short/long steps tend to follow long/short steps) or lack of memory (short and long steps alternate without any logic) in the observed dFC random walk, *Detrended Fluctuation Analysis* (DFA) is a tool of choice [39,50], as previously mentioned.

DFA infers a self-similarity coefficient by comparing the detrended mean square fluctuations of the integrated signal over a range of observation scales in a log-log plot (see examples in Fig. 2D). If the log-log plot has an extended linear section, (i.e. if the scaling relation is a genuine power-law over a reasonably broad and continuous range of scales, see later for the meaning of ‘genuine’), it means that fluctuations ‘look the same’ across different temporal scales, i.e. we have statistically the same fluctuations if we scale the intensity of the signal respecting the DFA exponent. The input to DFA analysis is, in our case, a dFC stream estimated along time in a way as smooth and continuous as possible. The first step is thus the evaluation of *instantaneous dFC increments*:

$$v_{\text{dFC},W}\left(t\right)=1-\text{dFC}\left(t,t+\delta t\right)$$

where δ*_t_* is the minimum possible sliding time-shift in our discretely sampled time-series (one data point of shift, i.e. one TR for fMRI time-series). Note that these instantaneous increments $v_{dFC,W}\left(t\right)$ continue to depend on the window-size W because the dFC streams are computed adopting a specific window-size. Therefore, a different fractal scaling analysis must still be performed for each of the different window sizes W. It may seem paradoxical to the adepts of fractal approaches that a scaling analysis still depends on the scale of the used estimation window. However, this can be mathematically understood by considering that what we are studying the scaling properties of a sequence of FC*(t)* matrices evaluated by a specific estimator (set by a choice of *W*) and not the scaling properties of the estimation itself of the FC*(t)* matrices. In other words, coarse graining is performed along the temporal dimension of the dFC temporal network, not along real time and we could obtain, in principle, a different temporal network for each choice of *W.*

To perform DFA, the time-series of instantaneous dFC increments is then converted into an unbounded process:

$$D_{W}\left(t_{i}\right)=\sum_{l=1}^{i}v_{dFC,W}\left(t_{l}\right)$$

Let K denote the number of samples in the time series, that are split into M non-overlapping segments q=1…M of length k each, with M=K/k. For each segment *q,* a fluctuation strength is computed as the squared difference between $D_{W}\left(t\right)$ and its trend $D_{W}^{\left(\text{trend}\right)}\left(t\right)$ (in the linear case this is the regression line of $D_{W}\left(t\right)$ over the interval t=1…k):

$$F_{q}^{2}\left(k\right)=\frac{1}{k}\sum_{l=0}^{k-1}{\left[D_{W}\left(t_{q+l}\right)-D_{W}^{\left(\text{trend}\right)}\left(t_{q+l}\right)\right]}^{2}$$

In the case of scale-free correlation this fluctuation strength scales with segment size k. That is, (on average) one finds a linear power law of the form:

$$\log\left(F_{\text{DFA}}\left(k\right)\right)=\log F_{q}\left(k\right)=\alpha_{\text{DFA}}\log k+C$$

The scaling parameter *α*_DFA_ is the primary outcome measure of DFA. In the case of the scale-free processes with the aforementioned power law, $\alpha_{\text{DFA}}$ resembles the Hurst exponent [50], leading to the interpretation:

• $0<\alpha_{\text{DFA}}<0.5$: $v_{dFC,W}\left(t\right)$ displays anti-persistent fluctuations
• $\alpha_{\text{DFA}}=0.5$: $v_{dFC,X}\left(t\right)$ displays uncorrelated Gaussian fluctuations (or, equivalently, *D_W_* resembles Brownian motion)
• $0.5<\alpha_{\text{DFA}}<1$: $v_{dFC,W}\left(t\right)$ displays persistent fluctuations (approaching “pink noise” when $\alpha_{\text{DFA}}$ is close to 1)
• $1\leq \alpha_{\text{DFA}}$: $v_{dFC,W}\left(t\right)$ is non-stationary (strictly speaking, DFA is undefined in this case)

In Battaglia et al. [5] we have found that resting state dFC streams are generally persistent (“pink noise” like, long-term correlations) for window sizes *W > ~*20 s, in line with frequent reports of persistence in neural activity [24,33]. This observation also reduces the importance of exactly optimizing the sliding window size chosen for dFC analyses. Indeed, as long as *W* is sufficiently large (e.g. in the order of a few tens of s), then, dFC random walks with qualitatively similar fractal geometry properties will be obtained. The relative stability of the *α*_DFA_ indicator makes it a candidate imaging-based marker to discriminate subject cohorts. For instance, we find that *α*_DFA_ exponents are generally closer to 0.5 and thus denoting more “randomness” and less complexity for elder subjects (cf. here Fig. 2D). Finally, even if *α*_DFA_ exponents must be evaluated separately for different windows, they could be averaged over different *W*’s with similar values of *α*_DFA_, or their distributions pooled for different *W’*s to reinstate a form of “smoothing by window pooling”. Such procedure once again is only pragmatic, not rigorous, and should be cross-checked by evaluating the stability of results for single window estimations over the considered range to pool.

In the dFCwalk toolbox, the function dFC_Speeds can be used to evaluate instantaneous dFC increments as well:

[∼,Increments]=dFC_Speeds(dFCstream2dFC(TS,W,1),1)

We also copy in the toolbox a function DFA_fun from an open MATLAB ^Ⓡ^ repository [35] for a fast “quick-and-dirty” evaluation of the DFA exponent. Such function can take as input the Increments variable generated by dFC_Speeds. However, for any serious research application beyond fast exploration we advise the use of a dedicated software solution for DFA analyses. Importantly, prior to construing outcome values (which can be always generated by a function even when they do not make sense), it is mandatory to verify that a linear power law scaling actually exists. If it was not the case indeed the output value $\alpha_{\text{DFA}}$ could not be interpreted as a scaling exponent. In Battaglia et al. [5] we followed Ton & Daffertshofer [46], testing the hypothesis of power-law scaling using a Bayesian model comparison approach and retaining only the subjects for which the DFA log-log plot was better fitted by a straight line than by any other tested alternative model. We refer the reader to Ton & Daffertshofer [46] for further guidance on how to proceed with quality DFA analyses.

### Meta-Connectivity analyses

**Fig. 3:**
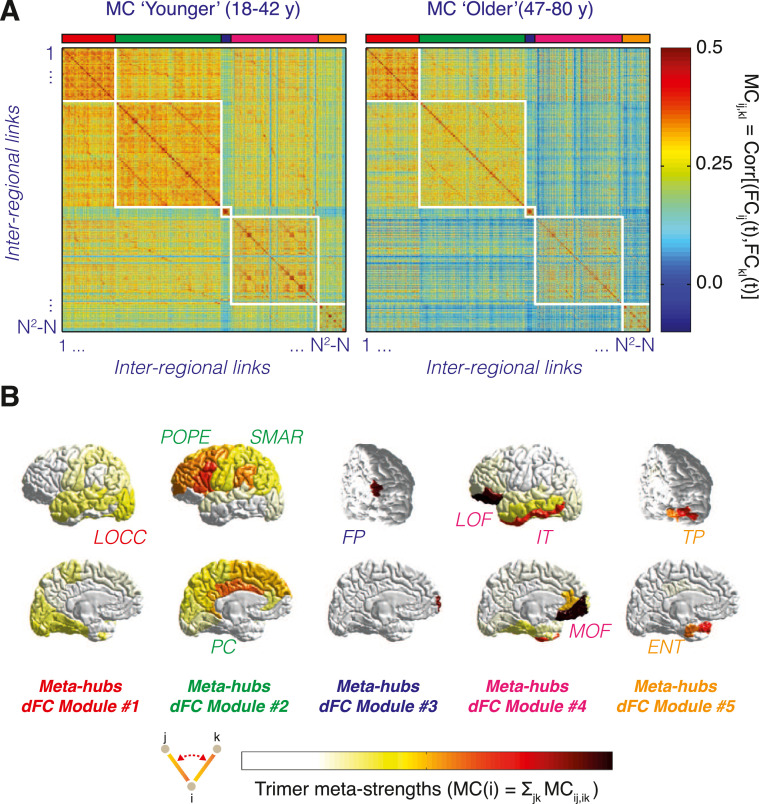
*Meta-Connectivity and dFC modules.* Correlations between the time-courses of pairs of inter-regional FC links are compiled into *Meta-Connectivity* (MC) matrices (cf. Fig. 1C). (A) Group-averaged MC for two distinct age groups (‘younger’: *N =* 42, 18–42 yrs, median age = 24 yrs; and ‘older’: *N =* 42, 47–80 yrs, median age = 63 yrs) based on smooth dFC streams (*W =* 30 s, ∆ = 1 TR). These matrices are evidently modular (after reordering of entries according to a standard Louvain algorithm). Groups of covarying links are called *dFC modules*. Differences in the inter-modular meta-connectivity between the ‘younger’ and the ‘older’ group appear visually evident. (B) Two meta-connected links converging on a common root node form what we call a *meta-connectivity trimer*. We call then *meta-hubs* nodes serving as root to many strong trimers (or, equivalently, displaying a strong trimer *meta-strength* MC(i)). In panel B brain regions –defined in a Desikan et al. [19] parcellation– are colored according to their meta-strength MC(*i*), restricted to each of the five dFC modules identified by the modular decomposition of the MC matrices in panel A. Many of these modules have meta-hubs resembling the ones of dFC modules discussed for a different dataset in Lombardo et al. [34].

Global dFC speed analyses consider whole-brain FC networks as a point in an abstract space. While such global analyses are suitable for studying *when* unspecified network reconfiguration occur, they do not allow identifying *where* these network changes are arising. Indeed, some groups of links may vary in a coordinated manner producing the waxing and waning of entire subnetworks, and these sets of covarying links may be organized around regions which act as control centers of ongoing FC network reconfiguration events. To study more in detail the spatiotemporal organization of ongoing dFC morphing we introduced an alternative characterization of the dFC stream. Each dFC stream can indeed be considered as a collection of time series describing the time-dependency of individual FC pairwise couplings (Fig. 1C). By extending the FC matrix construction from regional nodes to inter-regional links, an *M* × *M* matrix of correlations between the time-dependent strengths of *M = N(N*-1*)* FC links (*N^2^* pairs of regions, minus the self-loops) can be constructed. The resulting *Meta-Connectivity (MC)* matrix (see examples in Fig. 3A) describes thus inter-link covariance, similarly to FC describing inter-node covariance. MC matrices are particularly suitable for inter-group comparisons. In fact, they can be averaged over multiple subjects within a homogeneous group (expected to share common inter-link correlations), while this cannot be done for dFC matrices (portraying every time specific realizations of FC fluctuations, different for each subject in a group and even for each session within a given subject).

To compute MC, the starting point is a dFC stream. MC therefore also depends on the choice of a window size *W.* We advise the use of a short ∆τ to follow with smoothness link fluctuations and of a shorter *W* than what would be considered usually reasonable –in Lombardo et al. [34] we used e.g. *W* = ~20 s–, because the poorer precision in instantaneous inter-node correlations is compensated for by a larger number of non-overlapping time frames, needed for estimating inter-link correlations. Out of the dFC stream, we then extract the *n = N*(*N*-1)/2 time series of pairwise FC couplings, given by the entries FC*_ij_(t)* for all pairs of regions *i* and *j* with *i* < *j* ≤ *N.* Note that these time-series correspond to the *n* rows of the dFC stream computed by dFCstream2dFC in ‘vector’ format.The entries of the Meta-Connectivity matrix (MC) were then given by:

$$MC_{ij,kl}=\text{Corr}\left[FC_{ij}\left(t\right),FC_{kl}\left(t\right)\right]$$

As for the FC analysis, all MC entries are included into the matrix, independently of whether inter-link correlation values are significant or not or without fixing any threshold. These MC*_ij,kl_* entries are compiled into a matrix format, allowing the direct identification of the pair of links involved into each meta-link. Thus, MC matrices have a *M* × *M* size, where different rows correspond to different directed pairs of regions – i.e. both the pair (*i,j*) and the pair (*j,i*) were included – and only links corresponding to self-loops – i.e. of the type (*i,i*) – were excluded. The resulting MC representation is very redundant since:

$$MC_{ij,kl}=MC_{ji,kl}=MC_{ij,lk}=\ldots =MC_{kl,ij}=\ldots =MC_{lk,ji}$$

However, to be computationally efficient it is possible to just we compute Pearson correlations between pairs of time series FC*_ij_(t)* and FC*_kl_(t),* with *i* < *j, k < l, i ≤ k, j < l* and then copy these values into the eight degenerate MC matrix entries. Such redundancy allows interpreting MC as an adjacency matrix between links, so that conventional graph-theoretical analyses usually performed over networks of regional nodes can now be performed on meta-networks of inter-regional edges.

For instance, one can define the *meta-strength* of a node *i* as the sum of the meta-connectivity weights between all pairs of links incident on node *i*, i.e.:

$$\text{MC}\left(i\right)=\Sigma_{kl}MC_{ik,il}$$

A node with a particularly large meta-strength is termed a *meta-hub*. A meta-hub can be seen as the center of a star of incident links whose fluctuations are strongly covarying. We call the set of two links incident on a common root region a *trimer*, with the existence of inter-link covariance represented diagrammatically by a small spring between the meta-connected links (see Fig. 3B, bottom). A meta-hub can thus also be described as a node being the root of several strong trimers. Analogously we call *tetramers* the set of two non-incident links coupled by a meta-link. Usually, we have found that trimer strengths MC*_ik, il_* tend to be larger than tetramer strengths MC*_ij, kl_* with i≠j≠k≠l, which justifies paying particular attention to trimers and high (trimer) meta-strength nodes.

Note that MC is similar to other edge-based functional connectivity approaches introduced elsewhere [7,^10^,20]. In agreement with Braun et al. [7] and also Davison et al. [16,17] –further exploring similar methods–, we stress that MC captures higher-order correlations between triplets or quadruplets of brain regions, beyond pairwise FC. Indeed, estimating the strength of a meta-link MC*_ij,kl_* between two functional links FC*_ij_* and FC*_kl_* requires monitoring the coordinated activity of minimum three brain region (for MC trimers) or, generally, four (for MC tetramers). Thus, MC analysis tracks the static high-order correlation structures, which shape the coordinated stochastic fluctuations of second-order pairwise FC.

Unlike Brovelli et al. [10], conceived for applications to cognitive task analyses, our method does not require any alignment of trials to extrinsic cues for estimating time-dependent networks and is thus suitable to analysis of spontaneous dFC fluctuations, occurring not only along tasks but also in the resting state.

Furthermore, unlike the *edge-centric Functional Connectivity* (eFC) defined by Faskowitz et al. [20], MC profits of a sliding window smoothing and seems thus to be less sensitive to fast noise in link fluctuations [34]. We remind that, quite similarly to MC, eFC equally provides a description of correlations between link fluctuations. However, in the case of eFC, sliding windows are not used to estimate the dFC stream but correlation between link fluctuations are studied at the instantaneous level starting directly from raw time-series. The time-series BOLD*_i_*(*t*) are first z-scored, then time-series of pairwise products are constructed:

$$P_{ij}\left(t\right)=\text{zscore}\left[TS_{i}\left(t\right)\right]\cdot \text{zscore}\left[TS_{j}\left(t\right)\right].$$

Finally, eFC is constructed as the correlation matrix of these instantaneous pairwise product time-series:

$$\text{eF}C_{ij,kl}=\text{Corr}\left[P_{ij}\left(t\right),P_{kl}\left(t\right)\right]$$

As we show in Lombardo et al. [34] a strong correlation exists between MC and eFC matrix entries, although the two analyses are not exactly identical.

In the dFCwalk toolbox, we provide several functions useful for link covariance analyses. The function dFCstream2MC takes as input a dFC stream in either 2D or 3D formats and produce as output a *M* × *M* square MC matrix. The function Which_Link_ThisMCIndex allow feeding an integer 1 ≤ *m* ≤ *M* and obtaining the pair (*i,j*) of regions linked by the *m*-th edge, whose meta-link strengths are given by the *m-*th row in the MC matrix. The reverse information can be obtained by the function Which_MCIndex_ThisLink mapping a pair of regions *i* and *j* provided as input to the *m* ordinal associated to the link between them. We also provide a function TS2eFC that takes as input multivariate time-series of activity and gives back the associated *M* × *M* matrix of eFC. Finally, the function ExtractTrimerStrengths can take as input a MC matrix and restitute a vector with *N* entries corresponding to the meta-strengths of the *N* regional nodes.

### Modular and network-restricted dFC analyses

As previously said, the fact of packaging the meta-link strengths into a square MC matrix with large redundancy allows to treat it as if it was an ordinary network. Therefore, analyses such as conventional modularity analyses [11,41] can be applied to MC matrices using precisely the same algorithms adopted for FC networks. For instance, using the Louvain algorithm, or other community extraction algorithms will extract communities of regional nodes if applied to a FC network and communities of inter-regional functional edges if applied to a MC matrix. In conventional FC analyses, a FC module is a set of nodes whose activity fluctuations strongly covary with the activity fluctuations of adjacent nodes. In MC analyses, a *dFC module* (or meta-module) will be a set of functional links with covarying strengths, incident on a few meta-hubs, specific for the considered dFC module [34].

The aim is not here to explain methods for modular extraction (any method used elsewhere for community detection can readily be applied to MC). We present nevertheless an example of modular MC matrices to convince of the pertinence of performing modularity analyses on MC. Fig. 3A presents two group-averaged MC matrices obtained, respectively, from resting state fMRI on two cohorts of ‘younger’ (*N =* 42, 18–42 yrs, median age = 24 yrs) and ‘older’ (*N =* 42, 47–80 yrs, median age = 63 yrs) healthy subjects, analysed in Battaglia et al. [5] . See the original research article for details on these cohorts and on the resting state fMRI sessions. What is relevant here, first, is that MC matrices averaged over both these ensembles are clearly modular, as made visually evident by reordering the links according to their allegiance to 5 different meta-modules (extracted via a conventional Louvain algorithm). Second, differences between the MCs for the two groups are visually evident (with negative meta-link strengths emerging between the dFC modules in the MC for the ‘older’ group). Third, different dFC modules are centered around different meta-hubs (as visible by the meta-strength plots in Fig. 3B), distributed along a characteristic anteroposterior gradient. Remarkably the 5 dFC modules obtained here for the Battaglia et al. [5] datasets are remarkably reminiscent of the 5 dFC modules also identified in the datasets of Lombardo et al. [34] (notably the occipital, fronto-parietal and frontal modules have a good degree of overlap, although the used parcellations are different).

Once a modular partition of the MC matrix into dFC modules α, β, γ… (obtained with whatever method) is fixed, several analyses of the dFCwalk framework can be restricted to just one dFC module of interest. For instance, meta-strengths of regions can be computed by summing uniquely over meta-links between links belonging to a given dFC module α. Such restriction of the sum will provide dFC module-restricted meta-strengths:

$$MC^{\left(\alpha \right)}\left(i\right)=\Sigma_{kl|ik,il\in \alpha }MC_{ik,il}$$

To compute dFC module - restricted meta-strengths, the function ExtractTrimerStrengths can take as second optional input a list of the indices of the FC links included in the module of interest. For instance, if the Brain Connectivity Toolbox [41] is used to extract modules from MC via the function *C* = modularity_louvain_und_sign(MC) (note the use of a community extraction algorithm for signed and undirected graphs, since MC can be positive or negative and is symmetric), then the vector C will contain the *M* community labels (allegiance to a dFC module) for each one of the *M* links whose meta-connections are compiled into the MC matrix. The command Strengths_alpha = ExtractTrimerStrengths(MC, find(*C* == alpha)) will then return a vector with *N* dFC module restricted meta-strengths.

Any other dFC analysis (e.g. dFC speed or DFA) can be analogously restricted to a specific dFC module, by filtering in the dFC stream only the time-series associated to the included links. Let us suppose having a 2D or 3D format dFC stream variable dFCstream. Then the dFCwalk toolbox command SubgraphStream = Pick_SubgraphStream(dFCstream, links) will return a dFC stream restricted just to the links associated to the provided list of indices links. Any other function that can be applied to the original dFCstream can then also be applied to the restricted dFC stream. For instance:

[typSpeed,Speeds]=dFC_Speeds(Pick_SubgraphStream(dFCstream,find(C==alpha))

will compute the *modular dFC speeds* (see [34]) for the dFC module with label alpha. As discussed by Lombardo et al. [34], changes of modular dFC speeds may correlate with more specific and subtler variations of cognitive performance.

Finally, we stress that the list of links provided to ExtractTrimerStrengths or Pick_SubgraphStream in order to restrict analyses is not bound to correspond to a dFC module. As a matter of fact, it may correspond to whatever list of links of interest, such as the ones e.g. between a subset of regions in a specific anatomic subdivision (e.g. temporal lobe) or belonging to a classic network (e.g. default mode). The commands here introduced therefore can be used to extract generic network-restricted dFC analyses and not only dFC module-restricted quantities.

### Surrogate dFC streams

**Fig. 4:**
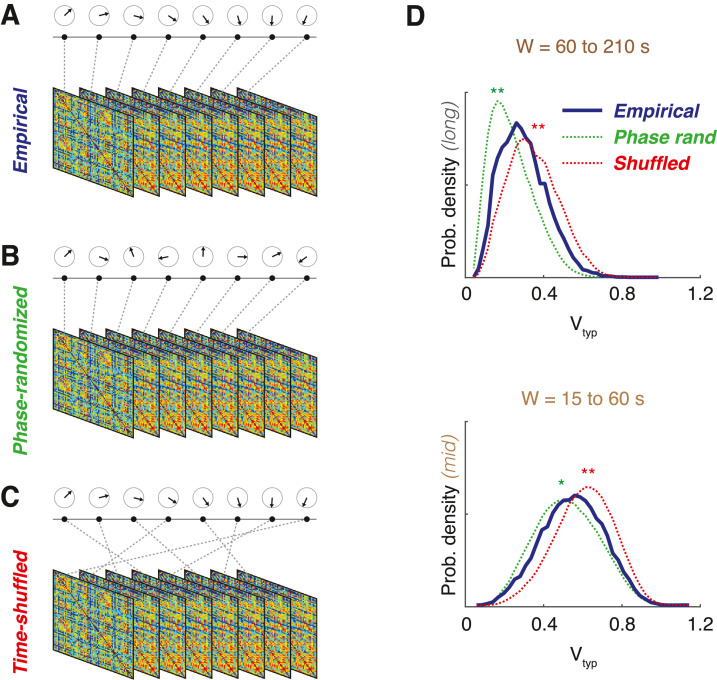
*Comparisons with surrogate dFC streams.* We compare dFC streams evaluated from actual empirical fMRI data (A) with surrogate dFC streams evaluated: from phase-randomized BOLD time-series surrogates (B), compatible with a null hypothesis of stationarity of the dFC stream; and from time-shuffled surrogates (C), compatible with an alternative null hypothesis of lack of sequential correlations in the dFC stream. (D) We also show for illustration distributions (smoothed kernel-density estimator) of typical resting-state dFC speeds for empirical and surrogate ensembles (distributions over grouped subjects), pooled over two distinct window-size ranges: long windows (top, 60 to 210 s) and intermediate windows (bottom, 15 to 60 s). Distributions for surrogate data significantly differ from distributions for empirical data (differences between empirical and time-shuffled distributions in red, between empirical and phase-randomized in green color; stars denote significant differences under two-sided Kolmogorov-Smirnov statistics: *, *p* < 0.05; **, p *<* 0.01).

It may be needed to assess statistically whether a certain dFC feature measured on an empirical dataset significantly differs from the value that such feature would be expected to assume under some null hypothesis. In order to test differences of empirical dFC streams (Fig. 4A) from chance expectations, it is important to define suitable surrogate dFC stream, corresponding to different possible null hypotheses, so that confidence intervals for null hypothesis expectations can be constructed by evaluating the feature of interest over a large number of surrogate dFC streams. In Battaglia et al. [5] we introduced two types of surrogate dFC streams, corresponding: either to fluctuations around an underlying “order” described by an unchanging, static FC (*FC stationarity*); or to a “disordered” complete lack of sequential correlations in the dFC stream (*temporally shuffled dFC).*

To probe the first “order” FC stationarity scenario, phase-randomized surrogates (Fig. 4B) can be constructed following Hindriks et al. [25]. In this approach, multi-variate time-series of BOLD signals are, first, Fourier transformed, extracting time-dependent amplitude and phases at different frequencies for every region. Then, random phase rotations are applied. Care must be taken in order to preserve not only the signal power spectra but also the overall multivariate signal average covariance matrix, as required for a null hypothesis of stationarity. Therefore, rather than applying independent random phase rotations to each of the regions, a common random phase rotation angle is generated at every time-step and applied to all the regions simultaneously, changing it step-by-step. This construction guarantees that the covariance matrix of the original empirical data is maintained but destroys any systematic deviation from stationarity. To perform these manipulations, MATLAB code kindly provided by the first author of Hindriks et al. [25] have been embedded in our dFCwalk toolbox and is accessible via the function PhaseRand_surrogates.

To probe then the second “disorder” scenario, one can construct time-shuffled surrogates (Fig. 4C). After constructing a dFC stream on the actual empirical data, a time-shuffled version of it can be computed by randomly permuting the order of the FC(t) timeframes but maintaining them individually unchanged. By this, the mean and variance of each of the FC connections independently are preserved but any sequential correlations are disrupted. Time-shuffled surrogates are thus compatible with a null hypothesis of absence of sequential correlations in the dFC stream. Standard MATLAB ^Ⓡ^ functions can be used to generate such time-shuffled surrogates, e.g. through the command ShuffledStream = dFCstream(:,:, randperm(1:*F*)) where *F* is the number of frames in the original empirical dFC stream.

We advise generating order 1000 instances of surrogate dFC stream to extract chance levels by taking 5th or 95th percentiles of the distribution of the feature of interest over these surrogate ensembles. In Fig. 4D, we show for instance comparisons between group distributions of typical dFC speeds for two different pooled window ranges, for empirical resting state fMRI vs both types of surrogate ensembles.

### Considerations on parameter choices

The presented dFC analyses give results dependent on parameter choices, as e.g. the choice of the window size *W*. Analogously, even if there are no strict preprocessing requirements for our toolbox to be used, the pre-processing steps applied to the time-series prior to analyses may affect the results obtained. Unfortunately there are no general rules that can be given, the choices to make depending on the specific problem, type of data and scientific questions.

Concerning signal smoothing for instance, often applied in resting state fMRI, it may contribute to the “liquid” appearance of dFC fluctuations. However, one should also consider that the time-constants of the haemodynamic response are rather slow and that we cannot expect fluctuations on scales faster than these time-constants to be due to genuine neural dynamics (since neural dynamics on these faster scales could not be translated into a sensible BOLD signal variation). Therefore, it would be reasonable to apply a low-pass filter to the data to cancel potential dFC fluctuations of non-neural origin. In Battaglia et al. [5] we applied to the fMRI time-series prior to dFC analyses a low-pass filter with a cutoff at 0.5 Hz, but this indication should not be taken as absolute and should be adapted for each study.

Concerning global regression, in principle it should be avoided because the global dFC fluctuations we want to capture with a dFC analysis would be certainly contributing to the global signal that global regression is meant to regress out (like throwing the baby out with the water). However, global regression may also be the only method available in certain case to compensate for unwanted artefacts of non-neural origin. The choice to apply or not global regression, at the end, will depend on the level of contamination of data by these unwanted artefacts.

Concerning the sampling rate of the data, the use of a larger sampler rate does not necessarily lead to more erratic and less smooth walks. Indeed, dFC walks are not the random walks of the multivariate signals themselves but the ones of their correlation which are still averaged and assessed over multiple points in the window. What could make the dFC walk more erratic is the use of a very short window size *W*. Even in this case, however, the increase in the number of temporal network frames may lead to a superior characterization of temporal network dynamics because more frames are available for dFC speed distribution and meta-connectivity estimation, thus improving their statistical estimation (noisier stream, but more observations). The choice of *W* in general will have to be optimized based on the signals sampling-rate and fluctuation properties.

Once again, there is no general rule-of-thumb. The researcher is advised to study the robustness of the results obtained for various choices of *W* and then select a *W* within a range of values for which the qualitative results don't change. The same applies to pre-processing choices, for which explorative dFC analyses with alternative pre-processing choices should be made at an early stage to become aware of the effects that different pre-processing may have on the interpretation of results.

### Scalability

The variables computed in dFC analyses can become very large and thus occupy a large amount of memory. This is particularly true for entities such as the dFC stream, a *N* × *N* × *F* tensor, or for the meta-connectivity, which is a *M* × *M* matrix. For instance, computing a dFC stream from a resting state session with *T* = 100 temporal images and *N* = 100 ROIs, adopting a small window size of *W* = 5 consecutive images (leading thus to a number of temporal frames in the stream equal to *F* = 996), leads to a dFC stream variable in three-dimensional tensor format (‘matrix’) of size 116 MB when saved in compressed binary format. Computing a meta-connectivity matrix out of this dFC stream would lead to a 9900 × 9900 matrix with memory occupancy of 651 MB.

This size is still reasonable but if the number of ROIs had raised to *N* = 1000, then the dFC stream would have raised to over 7 GB. The MC matrix would in this case become unfeasible on ordinary machines (in 2020), since its memory occupancy would exceed 7 TB.

Nevertheless, the dFCwalk toolbox provides (as default) the option to handle dFC analysis entities as the dFCstream in a compressed less redundant representation, as previously mentioned. For instance, the dFC stream once coded in ‘vector’ format becomes a *L* × *F* matrix, which has a memory occupancy reduced to the half.

Memory occupancy of dFC streams grows quadratically in the number of ROIs and linearly in the number of temporal frames. It grows with the fourth power in the number of ROIs for meta-connectivity matrices. This memory occupation caveats help defining research strategies. For short time-series with large number of ROIs (which is often the case of fMRI time-series with large parcellations or at the voxel level), meta-connectivity analyses may have prohibitive memory requirements, but dFC speed analyses based uniquely on the dFC stream more feasible. For very long-time-series (because of recording duration or very fine sampling rate, as it is often the case of EEG, MEG or LFP recordings), on the contrary, meta-connectivity may become a smaller entity than the dFC stream.

It is thus important to roughly estimate the memory size that an object can have before computing it, remarking that we store the individual entries of dFC entities in *double* format (8 bytes per element).

Time of computation will not be in general a limiting factor, since, for all the dFCwalk toolbox functions, it only grows at most linearly with the number of matrix or tensor entries to compute.

## Conclusion

We interpret dynamic Functional Connectivity (dFC) as a random walk in the space of possible FC networks performed with a quantifiable “speed”. Meta-connectivity provides a static representation of the spatial coordination structure underlying dynamic fluctuations of FC. A toolbox of “dFCwalk” functions naturally stems from our conceptual framework. We have illustrated the use of such a toolbox on resting state fMRI datasets, but our approaches can be applied to generic multivariate time-series of neural activity (or even to non-neural signals). The high sensitivity of dFC analyses, in particular in their module-restricted versions, bear promise of future applications to the early detection and longitudinal characterization of neuro-pathologies associated with cognitive impairment, such as Alzheimer's disease.

## Declaration of Competing Interest

The Authors confirm that there are no conflicts of interest.
